# Evaluation of Event-Related Potentials in Assessing Cognitive Functions of Adult Patients with Epilepsy of Unknown Etiology

**DOI:** 10.3390/jcm12072500

**Published:** 2023-03-25

**Authors:** Klaudia Jeżowska-Jurczyk, Piotr Jurczyk, Sławomir Budrewicz, Anna Pokryszko-Dragan

**Affiliations:** 1 Department of Neurology, Wroclaw Medical University, 50-556 Wroclaw, Poland; 2 Private Medical Practice, 52-207 Wroclaw, Poland

**Keywords:** epilepsy, cognitive impairment, event-related potentials, P300 potential

## Abstract

Background: Cognitive impairment (CI) is an important consequence of epilepsy. The aim of the study was to assess cognitive performance in patients with epilepsy, using neuropsychological tests (NT) and event-related potentials (ERPs), with regard to demographic and clinical data. Methods: The study comprised 50 patients with epilepsy of unknown etiology and 46 healthy controls. Based on the NT results, the patients were divided into subgroups with/without CI. Parameters of P300 potential were compared between the patients and controls. P300 parameters and NT results were referred to demographics and clinical characteristics of epilepsy. Results: Based on the NT, 66% of patients were assigned as cognitively impaired. Median P300 latency was significantly (*p* < 0.0002) prolonged in the study group. Subgroups of patients with and without CI significantly (*p* < 0.034) differed in education level and vocational activity, duration of epilepsy, age at its onset and frequency of polytherapy. P300 parameters showed significant (*p* < 0.03) relationships with duration of epilepsy, type and frequency of seizures and polytherapy. Conclusions: Cognitive impairment and ERPs abnormalities occur in a majority of patients with epilepsy of unknown etiology. Characteristics of epilepsy and socioeconomic status are related to cognitive performance. ERPs may complement neuropsychological methods in the assessment of cognition in patients with epilepsy.

## 1. Introduction

Epilepsy—a chronic condition characterized by a permanent predisposition towards developing seizures—constitutes a substantial clinical and social problem, due to its prevalence and specificity. Apart from the disease itself, somatic and mental consequences of epilepsy may significantly affect the patients’ quality of life [1]. Among these consequences, impaired cognitive performance deserves particular attention.

Cognitive impairment (CI) occurs in more than a half of patients with epilepsy. It is usually mild, although epilepsy is associated with double-increased risk of dementia [2]. The decline may be global or affect particular cognitive domains, most frequently affecting memory, verbal skills, attention, executive functions and visuospatial skills. CI is occasionally observed in the patients with newly diagnosed epilepsy; it may remain stable or gradually progress in the further course of the disease [3,4,5,6,7,8,9,10].

With recent progress in clinical research in epilepsy, a novel complex approach to its neurobehavioral phenotypes, including cognitive performance, has been developed [11]. This approach integrates biological factors (related to epilepsy, comorbidities and general health issues) with psychosocial and environmental ones. Depending on the background and type of disease, relevant epilepsy-related factors, potentially contributing to cognitive impairment, may include brain injury due to a range of disorders (e.g., vascular, degenerative, inflammatory), genetic mutations and variants, systemic metabolic alterations, as well as recurrent disturbances in cerebral bioelectrical activity and adverse effects of anti-epileptic treatment [3,9,11,12,13,14,15,16,17,18,19]. In the patients with epilepsy of unknown etiology, the majority of these factors have been eliminated, so the investigation of the background for CI may be focused on the functional impairment of neuronal networks. Thus, epilepsy of unknown etiology seems to provide a good model for the consistent and reliable analysis of epilepsy-related factors contributing to CI.

Better insight into these relationships and precise evaluation of cognitive performance might allow for identification of the patients with greater risk of CI and provide them with adequate support. According to the mentioned multidimensional neurobehavioral paradigm, resilience factors and cognitive reserve of these patients should be recognized, as well as socioeconomic status and resources. Based on these factors, complex therapeutic approaches might be elaborated, including optimal pharmacological treatment, modification of lifestyle factors and cognitive rehabilitation [11].

In the evaluation of cognitive performance, event-related potentials (ERPs) can be used alongside neuropsychological tests as its electrophysiological measure. ERPs represent an averaged response of cerebral bioelectrical activity to the series of stimuli, which are associated with a task requiring cognitive and emotional engagement of the subject. The most frequently assessed ERP component, P300 potential, reflects conscious information processing and decision making [20,21]. ERPs have been widely used in the evaluation of cognitive impairment in the course of various CNS disorders [22,23,24,25,26,27,28].

The aim of this study was to assess cognitive performance in the patients with epilepsy of unknown etiology, with the use of neuropsychological tests and event-related potentials (ERPs). We also aimed to analyze relationships between measures of cognitive performance and demographics and clinical characteristics of the disease.

## 2. Materials and Methods

Participants to the study were recruited from the patients diagnosed with epilepsy of unknown etiology, according to the International League Against Epilepsy criteria [29], with a documented course of the disease, who were hospitalized or consulted at the Department of Neurology, Wroclaw Medical University, in the years 2016–2019. Exclusion criteria included neuroimaging evidence of cerebral lesions corresponding with epileptic seizures or located in the areas potentially involved in cognitive functions, chronic and decompensated comorbidities which might affect cognitive performance, and mental disorders and/or severe cognitive impairment which would prevent providing an informed consent to participate in the study and performing neuropsychological tests involved in the study protocol.

Finally, the study group comprised 50 individuals: 44 women and 6 men, aged 20–68 years (mean—35.6). The control group consisted of 46 healthy volunteers, matched for age (23–57 years, mean—35.9) and sex (39 women and 7 men) to the study group.

The demographic data (age, sex, educational level, vocational activity) and information concerning epilepsy and coexisting diseases were initially obtained from a self-assessment questionnaire. The clinical data concerning epilepsy (age at onset, duration of the disease, type and frequency of seizures, current treatment) were then verified and completed on the basis of medical records, including the results of neuroimaging (magnetic resonance imaging—MRI or computed tomography—CT) performed within a previous year.

For the assessment of cognitive performance, the following tests were used: Rey Auditory Verbal Learning Test (AVLT), Rey–Osterrieth Complex Figure (ROCF), Trail Making Test (TMT), Digit Span Test from Wechsler Memory Scale (WMS-R), Similarities Test from Wechsler Adult Intelligence Scale (WAIS-R), Verbal Fluency Test (VFT)—for which utility and reliability in the assessment of patients with epilepsy had been proved in experiences of epileptological centers [30,31] (for details of the tests content, scoring and interpretation of results, see Appendix A). Based on the results of these tests, the study group was divided into two subgroups: with or without cognitive impairment (≥2 different tests failed vs. abnormal result of ≤1 test).

The examination of auditory event-related potentials was performed in the study group and with the healthy controls using a Nicolet 1000 Viking device, according to the guidelines from International Federation of Clinical Neurophysiology [32]. Surface electrodes were placed on the scalp at midline points Fz, Cz and Pz, according to a 10–20 system, with the reference electrode attached to linked earlobes and the ground one—to the forearm. Auditory stimuli (duration 200 ms, intensity 70 dB) were emitted binaurally through headphones using the ‘oddball paradigm’: 20% of target tones (frequency 2000 Hz) were randomly scattered among the remaining non-target ones (80%, frequency 1000 Hz). The subject’s task was to focus on target tones and count them silently in each series. The recording was performed with a bandpass filter of 0.3–70 Hz and sweep time of 1000 ms. With the use of dedicated software, the responses to target and non-target stimuli were averaged separately for each of the references, up to receiving at least 30 target responses. This procedure was performed twice for each subject. A P300 potential was identified in the target response curve for each reference as the highest positive component in range 250–500 ms. P300 latency (time since the occurrence of target stimulus to the peak of an identified potential) and amplitude (peak-to peak) were measured after each of the two recording series, and their final values were averaged from these.

In addition, patients with epilepsy had electroencephalography (EEG) performed, using a 24-channel Nicolet One device. Surface electrodes were placed on the scalp in standardized sites according to the international 10–20 system. EEG was recorded with a bandpass filter of 0.5–100 Hz, with a sampling frequency of 256 Hz and an analogue of 50 Hz filter. The subjects lied down and stayed awake with their eyes closed. During a 20 min recording, they were asked to breathe deeply for 3 min and, subsequently, photostimulation (flashes of stroboscopic light emitted with fluctuating frequency 4–30 Hz) was applied for 3 min. After the automatic rejection of artifacts, the EEG recording was visually inspected for any abnormal graphoelements, especially including paroxysmal epileptiform activity.

All the tests were performed during one session, in the morning hours. The date of session was established after at least 2 months since the introduction or recent modification of antiepileptic treatment, and at least 48 h after a recent seizure.

The study was conducted in accordance with the Helsinki Declaration. The design of the study and all the involved procedures were accepted by the Bioethics Committee at Wroclaw Medical University. All the participants had given their written, informed consent before they were included in the study.

The subgroups of patients with and without cognitive impairment were compared with regard to the demographics and characteristics of epilepsy. Results of ERPs were compared between the study group and controls. In the study group, ERP parameters were referred to demographic, clinical and neuropsychological data.

Mean averages (x), medians (M), ranges (min-max), lower and upper quartiles (25–75Q) and standard deviations (SD) of the recorded continuous parameters were calculated. Verification of the hypothesis of equal means parameters in independent groups was performed using the ANOVA variance analysis or with the Mann–Whitney nonparametric U test (for 2 groups) in two groups of heterogeneous variance or the Kruskal-Wallis test by ranks (for 3 and more groups)—homogeneity of variance was verified by Bartlett’s test. For discrete parameters, the incidence of characteristics in groups was analyzed with the χ^2^_df_ test with an appropriate number of degrees of freedom df (df = (m − 1) × (*n* − 1), where m—number of lines, *n*—number of columns). For chosen pairs of parameters, a correlation analysis using Spearman’s rank correlation coefficient was conducted. A multivariate analysis was performed, using logistic regression (quasi-Newton method) or backward multiple regression. *p* ≤ 0.05 was considered significant. Statistical analysis was performed using a digital packet of statistical software EPIINFO Ver. 7.2.3.1, developed by Centers for Disease Control and Prevention (CDC) in Atlanta, Georgia (US).

## 3. Results

### 3.1. Demographic Data

The study group and controls did not differ significantly in age (*p* = 0.632) or sex (χ^2^ = 0.212, *p* = 0.645). Among the patients with epilepsy, 13 persons (26%) had completed primary or vocational school, 18 (36%)—secondary school and 19 (38%)—university degree. A total of 31 patients (62%) were full-time or part-time employed.

### 3.2. Clinical Data

#### 3.2.1. Course of Epilepsy

Disease duration in the study group ranged between 0.4 and 41 years (mean: 15 years) and age at onset—from 2 to 52 years (mean 21). Types and frequency of epileptic seizures are listed in Table 1. Family history of epilepsy was positive in six patients (12%). All the subjects were receiving antiepileptic treatment: 18 (36%)—monotherapy, 32 (64%)—polytherapy (most frequently with levetiracetam (*n* = 21) and lamotrigine (*n* = 18)) (Table 1).

#### 3.2.2. Neuroimaging

Within the previous year, an MRI was performed in 46 patients (92%), and a CT in 4 patients (due to contraindications or lack of the subjects’ consent to perform MRI). All CT scans and 38 MRI scans showed no structural brain abnormalities. In eight cases, a few small hyperintensive lesions were revealed in cerebral deep white matter, which did not correspond with type of seizures, and were outside of areas strategic for cognitive functions, and were considered as clinically non-significant.

#### 3.2.3. EEG

EEG recording was normal in 26 patients (52%), while in 24 patients interictal paroxysmal epileptiform activity was found (in 8 cases accompanied by the slowing of background activity) (Table 1). Out of these, paroxysmal epileptiform activity was recorded in 14 patients only during activation procedures (hyperventilation and/or photostimulation). None of the subjects experienced an epileptic seizure during or after EEG recording.

#### 3.2.4. Coexisting Disease

Concomitant somatic disorders were documented in 13 (26%) patients—arterial hypertension (*n* = 5), dyslipidemia (*n* = 5), diabetes, polycystic ovary syndrome and psoriasis (in a single person each).

Twelve (24%) patients had been diagnosed with mental disorders: in five cases (10%)—depression, in two (4%)—anxiety disorder and in five (10%)—dissociative disorder. Those diagnosed with depression were undergoing pharmacological treatment and others—psychotherapy.

### 3.3. Neuropsychological Assessment

The results of all neuropsychological tests conducted in the study group are listed in Table 2. The highest percentage of abnormal results was received for AVLT (verbal memory, learning ability) and TMT (attention, executive and visuospatial functions, psychomotor speed) and the lowest—for WAIS-R Similarities Test (abstract and associative thinking) (Table 2). The number of abnormal results scored by each individual in the study group ranged from 0 to 10 (mean 3.1 ± 2.5).

On the basis of these results, the study group was divided into subgroups without (abnormal result of ≤1 test) or with (≥2 different tests failed) CI.

#### Neuropsychological Assessment vs. Demographic and Clinical Data

The subgroups without CI and with CI did not differ significantly for age and sex. The subgroups without CI presented with a higher frequency of professional activity, and even more significantly, a higher level of education (Table 3).

As for clinical data, the subgroup without CI showed (with high significance level) a later age of onset and shorter duration of epilepsy, and a lower proportion of patients on polytherapy (Table 3). No differences were found between the subgroups for type and frequency of epileptic seizures, or the presence of abnormalities in EEG recording. 

After a multivariate analysis using logistic regression, an independent significant relationship was found between cognitive impairment and duration of epilepsy (lasting more than 7 years) (χ^2^ = 14,6; *p* = 0.00013).

### 3.4. Event-Related Potentials

**Figure 1:**
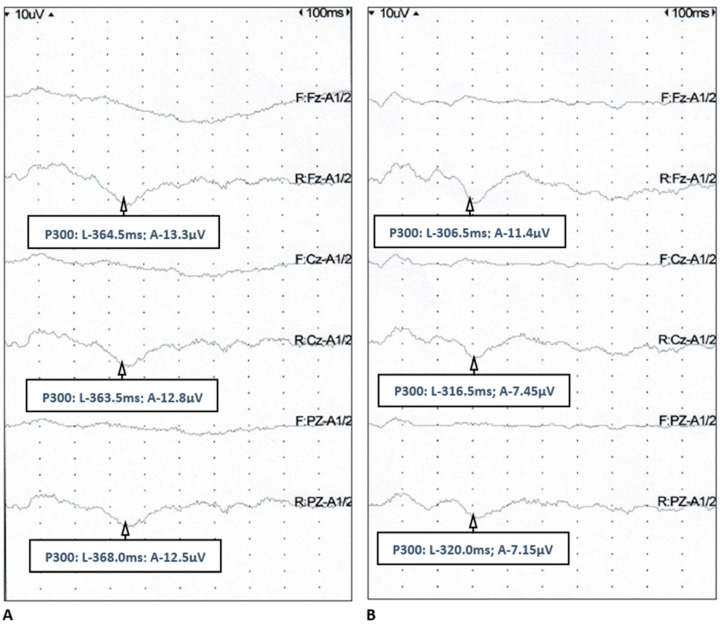
P300 event-related potential: in a 28-year-old woman with epilepsy (**A**) and a 30-year-old woman from the control group (**B**) (L—latency, A—amplitude).

The P300 component was identified in ERPs recorded from 44 patients with epilepsy (88%) and all healthy controls. Median latency of P300 in all the references was significantly longer (with high significance) in the study group than in the controls, whereas median amplitude of P300 did not differ between the groups (Table 4, Figure 1).

#### 3.4.1. Event-Related Potentials vs. Demographic and Clinical Data

In the study group, a significant positive correlation was observed between age and P300 latency but only for the Pz reference (R = 0.32; *p* = 0.0363). Because of a small proportion of male subjects in the study group, no relationships were analyzed between P300 parameters and sex.

With regard to clinical characteristics of epilepsy, a moderately significant positive correlation was found among P300 latency in all the references (especially in Cz and Pz) and duration of disease (Table 5). Median P300 latency was longer (moderate significance) in the patients with focal seizures (in comparison with generalized ones) and—even more significantly—in those on polytherapy (Table 6 and Table 7). Median P300 amplitude in Fz was the highest in the patients with monthly occurrence of seizures and the lowest—in those with weekly occurrence (M = 7.2 vs. M = 3.65, *p* = 0.0177). Median P300 amplitude in Cz was lower in the patients with paroxysmal activity in EEG than in those with normal EEG (M = 5.78 vs. M = 9.33, *p* = 0.00101).

#### 3.4.2. Event-Related Potentials vs. Neuropsychological Assessment

In the overall comparative analysis, no significant differences in P300 parameters were found between the subgroups of patients with and without cognitive impairment (Table 8). In the search for relationships between the P300 parameters and particular test results, we demonstrated the most consistent significant negative correlations between P300 latency and the results of AVLT (total and after delay), WAIS-R Similarities Subscale and (with lower significance level) semantic VFT. Negative correlations were also found between P300 amplitude and the results of TMT A and B, but only for Cz reference (Table 9).

## 4. Discussion

Neuropsychological assessment revealed cognitive impairment in more than 60% of the study group of patients with epilepsy. The most commonly affected domains included verbal memory and learning abilities, attention, psychomotor speed, visuospatial and executive functions, while performance in nonverbal memory and abstract thinking often remained preserved. Other reports in this field, though diverse in methodology, show similar incidence and profile of cognitive impairment in the patients with various types of epilepsy [3,4,9,10,14,33,34].

The P300 component of ERPs is generated in cortico–subcortical structures (including hippocampus, thalamus and (pre)frontal areas), which are involved in cognitive processes [35,36,37]. P300 latency is interpreted as the index of time necessary for information processing and problem solving, whereas the amplitude reflects the subjects’ engagement and focusing on the task. In our study, P300 latency was significantly prolonged in the patients with epilepsy in comparison with the healthy controls, while no differences were found for P300 amplitude. Increases in P300 latency and—less frequently—lowering of P300 amplitude were reported in several studies on epilepsy [38,39,40,41,42,43,44,45,46,47,48,49,50,51,52,53,54,55,56,57,58].

Although P300 parameters are usually considered as indices of global cognitive performance, the main processes involved in generating P300 comprise attention, executive functions, psychomotor speed and (to a lesser extent) memory [59,60]. In the current study significant relationships were found mostly between P300 latency and results of the tests evaluating verbal memory, abstractive/associative thinking and executive functions with semantic verbal fluency. These correlations seem particularly interesting, considering the type of task used to evoke auditory P300 (‘oddball paradigm’ with tones of different frequency). Furthermore, P300 parameters did not differ between the subgroups of patients with or without CI. It seems that P300 does not correspond with performance in specific cognitive domains, but rather reflects more complex interactions within cognitive skills. Thus, electrophysiological and neuropsychological measures of cognitive performance should not be considered as convertible but rather as complementary ones [61].

Except for correlation of P300 latency with age, no relationships were found in the study group between age and cognitive performance. Indeed, P300 latency increases with age [62]. However, this correlation was limited only to one reference, and the patients did not differ in age from the control group, so the prolongation of P300 latency in the study group remains relevant. Subgroups with and without CI did not differ in sex structure. However, a marked sex imbalance with underrepresentation of men (6/44) in the study group prevented the reliable analysis of relationships between sex and cognitive performance.

Our patients with cognitive impairment had a lower level of education and were less frequently employed. These findings, consistent with the results from the other studies [15,63,64,65], highlight the importance of cognitive performance in social functioning of the patients with epilepsy. Low level of education and limited cognitive reserve may predispose individuals to the development of cognitive impairment in the course of the disease, but also early cognitive deficit, emerging from epilepsy onset at a young age, may impede continuing education [66]. The same perspective, affected by the role of socioeconomic and cultural background, can be referred to the vocational activity of patients [67]. In our study, a percentage of the subjects who were not employed was relatively high, in comparison to the data from other European countries [16,68]. Educational and vocational issues, socioeconomic status and availability of social support are substantially linked with neurobehavioral aspects of epilepsy, as well as the patients’ quality of life [11].

Among epilepsy-related clinical data, the duration of the disease showed most significant relationships both with P300 parameters and findings from neuropsychological assessment, with the latter also associated with age at onset of epilepsy. An impact of these factors upon cognitive performance in epilepsy has been already reported [4,14,34,65]. Long-term dysfunction of neuronal network plasticity, caused by recurring seizure activity, is supposed to be the major contributor to cognitive impairment. According to a cascade model, onset of these disturbances at the early stage of intellectual development plays a crucial role in further cognitive decline [12,15,69]. Some links were noted in the study group between P300 amplitude and frequency of seizures, but only for the single reference, which limits their significance. We also found increased P300 latency in the patients with focal seizures in comparison with those with generalized ones. Focal seizures are associated with the local disintegration of neuronal network activity which may be relevant for the structures that are strategic for cognitive processes. Considering the moderate sample size, we did not distinguish subjects with particular localization of focal seizures (e.g., frontal or temporal ones) for further analysis. Moreover, P300 parameters are considered as indices of global bioelectrical event-related activity [35,36,37]. It should be highlighted that a diversity of epilepsy-related data in the study group might have affected the findings. Other studies investigating P300 parameters with regard to clinical characteristics of epilepsy showed inconsistent results [38,39,40,43,44,46,47,48,51,52,54,55,58,70].

Polytherapy was another factor significantly related to neuropsychological test results and P300 parameters in the study group. Basically, treatment reduces seizure frequency and stabilizes the bioelectrical activity of the brain, potentially preventing cognitive decline; but on the other hand—adverse effects of antiepileptics (enhanced by their interactions) may include cognitive dysfunction. The majority of studies reveal mild or moderate adverse impact of polytherapy on cognition [14,15,34,64,71], and on alterations of evoked potentials’ parameters (ERP, EP—high frequency oscillations) [39,43,46,55,72]. In the analysis of the particular antiepileptic medication effects in this field, one should consider their mode of action (modulation of synapses excitability via ion channels), as well as extent of functionally impaired cortico–subcortical circuits which underlie specific types of seizures and are involved in the generation of evoked potentials’ components [73,74].

On the analysis of relationships between cognitive performance and epilepsy characteristics, the interrelations among the latter and their accumulated effect have to be considered. In the study group, the patients with focal epilepsy had longer disease duration and higher frequency of seizures, and more often were being treated with polytherapy.

Overall, identification of these patients burdened with the factors predisposing to CI should encourage thorough assessment of their cognitive performance (e.g., with the use of neuropsychological and electrophysiological measures), followed by appropriate interventions (cognitive rehabilitation, optimization of treatment, psychological support).

### Strengths and Limitations of the Study

Our study attempted a comprehensive analysis of cognitive performance in the patients with epilepsy, with regard to demographic and clinical data. Integration of neuropsychological and electrophysiological findings provided more detailed insight into the prevalence and profile of cognitive impairment in the study group. The focus on patients with epilepsy of unknown etiology, due to the limited range of factors able to affect cognitive functions, allowed a more consistent and reliable analysis of relationships between them.

The limitations of the study included its cross-sectional mode, relatively small sample size and diversity of epilepsy characteristics, despite the aforementioned exclusion criteria. Considering the complex relationships between CI and education in epileptic patients, the differences in level of education between the study subgroups might be an additional source of bias. Nevertheless, the obtained results highlight a need for the precise evaluation of cognitive performance and considering this aspect in the complex approach to the management of epilepsy. There is a need for future studies on this topic require a comprehensive model to account for the multidimensional nature of the problem, as it is presently difficult to characterize how different factors may affect one another. Further investigation in this field seems necessary, comprising patients with different types of epilepsy, and monitoring cognitive deficit throughout the course of disease, to determine therapeutic implications of neuropsychological and electrophysiological findings.

## 5. Conclusions

Cognitive impairment and abnormalities of ERPs occur in a vast majority of patients with epilepsy of unknown etiology. Characteristics of epilepsy and socioeconomic status are potential factors related to cognitive performance. Event-related potentials may complement neuropsychological methods in the assessment of cognition, which constitute important elements in a multidimensional approach to epilepsy management.

## Figures and Tables

**Table 1:** Characteristics of epilepsy in the study group.

		Number of Patients (%)
Type of seizures	Generalized onset (tonic–clonic)	23 (46%)
	Focal onset	27 (54%)
	Impaired awareness	24 (89%)
	To bilateral tonic–clonic	20 (74%)
Frequency of seizures	>1/week	17 (34%)
	1/week- 1/month	15 (30%)
	1/month- 1/year	8 (16%)
	<1/year	10 (20%)
Antiepileptic medications	Monotherapy:	18 (36%)
	LTG ^1^	10 (56%)
	CBZ ^2^	3 (17%)
	LEV ^3^	3 (17%)
	TPM ^4^	1 (6%)
	VPA ^5^	1 (6%)
	Polytherapy 2 medications:	21 (42%)
	LEV ^3^ +LTG ^1^	5 (24%)
	LTG ^1^ +VPA ^5^	4 (19%)
	LEV ^3^ +TPM ^4^	3 (14%)
	LEV ^3^ +VPA ^5^	3 (14%)
	CBZ ^2^ +LEV ^3^	2 (10%)
	CBZ ^2^ +LTG ^1^	1 (5%)
	CBZ ^2^ +VPA ^5^	1 (5%)
	LEV ^3^ +PGB ^6^	1 (5%)
	LTG ^1^ +TPM ^4^	1 (5%)
	Polytherapy 3 medications:	11 (22%)
	LCM ^7^ +LEV ^3^ +TPM ^4^	2 (18%)
	CBZ ^2^ +GBP ^8^ +LCM ^7^	1 (9%)
	CBZ ^2^ +LEV ^3^ +LTG ^1^	1 (9%)
	CBZ ^2^ +LTG ^1^ +TPM ^4^	1 (9%)
	CZP ^9^ +LEV ^3^ +LTG ^1^	1 (9%)
	LCM ^7^ +LTG ^1^ +TPM ^4^	1 (9%)
	LEV ^3^ +LTG ^1^ +TPM ^4^	1 (9%)
	LEV ^3^ +LTG ^1^ +VPA ^5^	1 (9%)
	LEV ^3^ +TGB ^10^ +VPA ^5^	1 (9%)
	LTG ^1^ +OXC ^11^ +VGB ^12^	1 (9%)
EEG	Normal	26 (52%)
	Epileptiform activity	24 (48%)

^1^ LTG—lamotrigine; ^2^ CBZ—carbamazepine; ^3^ LEV—levetiracetam; ^4^ TPM—topiramate; ^5^ VPA—valproic acid; ^6^ PGB—pregabalin; ^7^ LCM—lacosamide; ^8^ GBP—gabapentin; ^9^ CZP—clonazepam; ^10^ TGB—tiagabine; ^11^ OXC—oxcarbazepine; ^12^ VGB—vigabatrin.

**Table 2:** Results of neuropsychological tests in the study group (SD—standard deviation).

TEST	Mean ± SD	Number (Percentage) of Abnormal Results
AVLT		
AVLT total	45.4 ± 10.5	22 (44%)
AVLT after distraction	8.6 ± 3.5	23 (46%)
AVLT delayed	8.0 ± 3.4	24 (48%)
ROCF		
ROCF copying	33.3 ± 5.6	7 (14%)
ROCF recall	16.1 ± 7.1	13 (26%)
TMT		
TMT A	51.3 ± 60.5	20 (40%)
TMT B	110.2 ± 104.0	21 (42%)
WAIS-R		
WMS-R Digit Span Test	10.8 ± 4.3	7 (14%)
WAIS-R Similarities Test	14.1 ± 5.4	1 (2%)
VFT		
Phonetic	13.0 ± 5.6	-
Semantic	17.7 ± 6.6	(38%)

**Table 3:** Comparison of subgroups without cognitive impairment (1) and with cognitive impairment (2) with regard to demographic and clinical factors (25Q–25th quartile; M—median; 75Q–75th quartile).

Subgroups	Without CI Z	With CI	
Sample Size	17	33	
Age			*p* = 0.229 *
25Q	26	29	
M	33	36	
75Q	37	43	
Sex			*p* = 0.378 χ^2^ = 0.778
Women	14 (82%)	30 (91%)	
Men	3 (18%)	3 (9%)	
Education			*p* = 0.00300 χ^2^_2_ = 11.6
Primary/vocational	2 (12%)	11 (33%)	
Secondary	3 (18%)	15 (46%)	
Higher	12 (70%)	7 (21%)	
Professional activity			*p* = 0.0333 χ^2^ = 4.53
Active	14 (82%)	17 (52%)	
Non-active	3 (18%)	16 (48%)	
Duration of epilepsy (years)			*p* = 0.00019 *
25Q	2	9	
M	7	17	
75Q	10	25	
Age of onset (years)			*p* = 0.00163 *
25Q	19	12	
M	25	16	
75Q	29	22	
Polytherapy	6 (35%)	26 (79%)	*p* = 0.00240 χ^2^ = 9.21

* a Mann–Whitney nonparametric U test was used.

**Table 4:** Comparison between P300 parameters in the study group and control group (25Q—25th quartile; M—median; 75Q—75th quartile).

	Study Group (*n* = 44)			Control Group (*n* = 46)			
	25Q	M	75Q	25Q	M	75Q	
P300 latency (ms)							
Fz	316.8	356.8	370.0	305.0	321.0	335.0	*p* = 0.00005
Cz	315.0	355.3	367.8	304.0	324.0	335.0	*p* = 0.00019
Pz	322.5	354.0	369.0	305.0	322.5	339.0	*p* = 0.00004
P300 amplitude (mV)							
Fz	3.68	5.40	8.35	4.00	6.73	10.00	*p* = 0.218
Cz	5.18	6.93	9.83	4.95	7.35	10.00	*p* = 0.449
Pz	6.55	8.20	11.25	5.80	8.15	11.60	*p* = 0.882

**Table 5:** Analysis of correlation between P300 parameters and duration of epilepsy in the study group (R—Spearman’s rank correlation coefficient).

	Duration of Epilepsy	
P300 latency (ms)		
Fz	*p* = 0.0116	R = 0.38
Cz	*p* = 0.00627	R = 0.41
Pz	*p* = 0.00708	R = 0.40
P300 amplitude (mV)		
Fz	*p* = 0.290	R = −0.16
Cz	*p* = 0.196	R = −0.20
Pz	*p* = 0.519	R = −0.10

**Table 6:** Analysis of correlation between P300 parameters in the study group and the type of epilepsy (25Q–25th quartile; M—median; 75Q–75th quartile).

Type of Epilepsy	Focal			Generalized			
	25Q	M	75Q	25Q	M	75Q	
P300 latency (ms)							
Fz	322.5	362.3	391.5	306.5	349	360	*p* = 0.0255
Cz	327	359.5	391.5	310.5	345.8	359.5	*p* = 0.0272
Pz	337	358	389.5	313.5	347.8	357	*p* = 0.00427
P300 amplitude (mV)							
Fz	3.65	5.03	7.65	4	5.58	10.1	*p* = 0.357
Cz	5.7	7.4	9.4	4.8	6.75	11.25	*p* = 0.917
Pz	6.7	8.68	10.7	6.4	8	12.85	*p* = 0.789

**Table 7:** Analysis of correlation between P300 parameters in the study group and type of therapy (25Q–25th quartile; M—median; 75Q–75th quartile).

Type of Therapy	Monotherapy			Polytherapy			
	25Q	M	75Q	25Q	M	75Q	
P300 latency (ms)							
Fz	306.5	320.3	355	353	362.3	377	*p* = 0.00997
Cz	310.5	319.5	349	355	360	373.5	*p* = 0.00498
Pz	316	327	352	353	358	370.5	*p* = 0.00796
P300 amplitude (mV)							
Fz	4.9	6.35	10.25	3.4	5.18	7.65	*p* = 0.0880
Cz	5.3	7.78	11.25	5.05	6.5	9.25	*p* = 0.204
Pz	5.5	8.85	13.6	6.7	7.95	10.2	*p* = 0.401

**Table 8:** Comparison of P300 parameters between the subgroups without (1) and with (2) cognitive impairment (25Q–25th quartile; M—median; 75Q–75th quartile).

	Subgroup 1 (*n* = 16)			Subgroup 2 (*n* = 28)			
	25Q	M	75Q	25Q	M	75Q	
P300 latency (ms)							
Fz	308.0	333.8	360.3	325.3	359.5	373.8	*p* = 0.0651
Cz	308.8	332.0	357.8	325.0	359.0	371.8	*p* = 0.0651
Pz	318.3	338.0	354.0	329.5	358.0	370.3	*p* = 0.0810
P300 amplitude (mV)							
Fz	4.95	7.25	9.83	3.53	5.18	7.90	*p* = 0.211
Cz	5.73	9.65	11.38	4.95	6.50	7.85	*p* = 0.0810
Pz	5.40	10.50	14.85	6.90	7.75	9.53	*p* = 0.186

**Table 9:** Correlations between P300 parameters and neuropsychological test results in the study group (R—Spearman’s rank correlation coefficient).

	P300 Latency			P300 Amplitude		
	Fz	Cz	Pz	Fz	Cz	Pz
AVLT						
AVLT total	*p* = 0.00667	*p* = 0.0152	*p* = 0.0179	*p* = 0.526	*p* = 0.109	*p* = 0.222
	R = −0.40	R = −0.36	R = −0.36	R = 0.10	R = 0.24	R = 0.19
AVLT after distraction	*p* = 0.0594	*p* = 0.134	*p* = 0.129	*p* = 0.332	*p* = 0.172	*p* = 0.452
	R = −0.29	R = −0.23	R = −0.23	R = 0.15	R = 0.21	R = 0.12
AVLT after delay	*p* = 0.00736	*p* = 0.0215	*p* = 0.0361	*p* = 0.397	*p* = 0.0886	*p* = 0.563
	R = −0.40	R = −0.35	R = −0.32	R = 0.13	R = 0.26	R = 0.09
ROCF						
ROCF copying	*p* = 0.268	*p* = 0.261	*p* = 0.419	*p* = 0.960	*p* = 0.128	*p* = 0.351
	R = −0.17	R = −0.17	R = −0.13	R = −0.01	R = 0.23	R = 0.14
ROCF drawing	*p* = 0.564	*p* = 0.633	*p* = 0.504	*p* = 0.646	*p* = 0.545	*p* = 0.568
	R = −0.09	R = −0.07	R = −0.10	R = 0.07	R = 0.09	R = 0.09
TMT						
TMT A	*p* = 0.188	*p* = 0.107	*p* = 0.160	*p* = 0.345	*p* = 0.0239	*p* = 0.124
	R = 0.20	R = 0.25	R = 0.22	R = −0.15	R = −0.34	R = −0.24
TMT B	*p* = 0.106	*p* = 0.114	*p* = 0.135	*p* = 0.268	*p* = 0.0120	*p* = 0.169
	R = 0.25	R = 0.24	R = 0.23	R = −0.17	R = −0.38	R = −0.21
WAIS-R						
WMS-R Digit Span Subtest	*p* = 0.558	*p* = 0.748	*p* = 0.707	*p* = 0.411	*p* = 0.105	*p* = 0.251
	R = −0.09	R = −0.05	R = −0.06	R = 0.13	R = 0.25	R = 0.18
WAIS-R Similarities Subscale	*p* = 0.0211	*p* = 0.0306	*p* = 0.0353	*p* = 0.958	*p* = 0.306	*p* = 0.682
	R = −0.35	R = −0.33	R = −0.32	R = −0.01	R = 0.16	R = 0.06
VFT						
Phonetic fluency	*p* = 0.0867	*p* = 0.141	*p* = 0.116	*p* = 0.967	*p* = 0.677	*p* = 0.562
	R = −0.26	R = −0.23	R = −0.24	R = 0.01	R = 0.06	R = −0.09
Semantic fluency	*p* = 0.0437	*p* = 0.0660	*p* = 0.0449	*p* = 0.637	*p* = 0.259	*p* = 0.596
	R = −0.31	R = −0.28	R = −0.30	R = 0.07	R = 0.17	R = 0.08

## Data Availability

Publicly available datasets were analyzed in this study. This data can be found here: https://ppm.umed.wroc.pl/info/achievement/UMWb9153c4b510e4a0e95b9aeee01b61cf1/ (accessed on 17 June 2021).

## Appendix A. Neuropsychological Test Used for Assessment of Cognitive Performance

• Rey Auditory Verbal Learning Test—AVLT

The test consists of five presentations of a list of 15 words (list A). After each reading, the participant is asked to recall as many words as possible. Next, the investigator presents another list of 15 words (list B). The participant is asked to recall the words from list B, then again from list A. After 20 min, the participant recalls words from list A once again. The results include the number of words from list A remembered in the first part of the test, after a distraction (list B) and after a delay. The test evaluates verbal memory and learning ability [75].

• Rey–Osterrieth Complex Figure—ROCF

The participant’s task is to copy a figure from a provided pattern, and then recreate it after 3 min in an Immediate Recall trial. Accuracy in copying and correct placement of all 18 elements of the figure are graded in both trials. The test assesses nonverbal memory, attention, executive and visuospatial skills [76].

• Trail Making Test—TMT

Part A of the test includes drawing lines linking 25 circles labeled with subsequent numbers. In part B, the subject is asked to alternately link circles labeled with numbers and letters (1-A-2-B-3-C, etc.). The result is the time needed to complete each part. The test assesses attention, executive and visuospatial skills and psychomotor speed [77].

• Digit Span Test from Wechsler Memory Scale—WMS-R

In the first part of the test, the participant repeats a series of numbers in an order given by the investigator, whereas in the second part—repeats them backwards. The result is a sum of correct answers. The test evaluates verbal memory and attention [78].

• Similarities Test from Wechsler Adult Intelligence Scale—WAIS-R

The participant is asked to find similarities/common features of sequentially presented pairs of words and can receive 0–2 points, depending on the correctness and precision of the answer. The result is a sum of received points. Abstract and associative thinking are assessed [78].

• Verbal Fluency Test—VFT

The task includes listing possibly many words beginning with the letter ‘k’ (phonetic fluency) and then names of animals (semantic fluency) within 60 s each. The result equals the number of words fulfilling the criteria of each task. The test assesses verbal fluency [79].

The results of the tests were then referred to standardized norms and considered abnormal if they exceeded 1 standard deviation (SD) (AVLT, ROCF, TMT, VFT) or 2 SDs (WAIS-R) with regard to age-adjusted normative values [78,80,81].
